# Supplementary material for: Optimization of differentiation and transcriptomic profile of THP-1 cells into macrophage by PMA
Source: PLoS One. 2023 Jul 17;18(7):e0286056. doi: 10.1371/journal.pone.0286056 (PMC10351730; doi:10.1371/journal.pone.0286056)
Supplement: S1 File — (ZIP) [file pone.0286056.s001.zip › supporting files/RNA-seq report.zip!/RNA-seq report/4_GOEnrichment_result/induce_vs_control/DAG/induce_vs_control.DEG_cc_DAG.pdf]

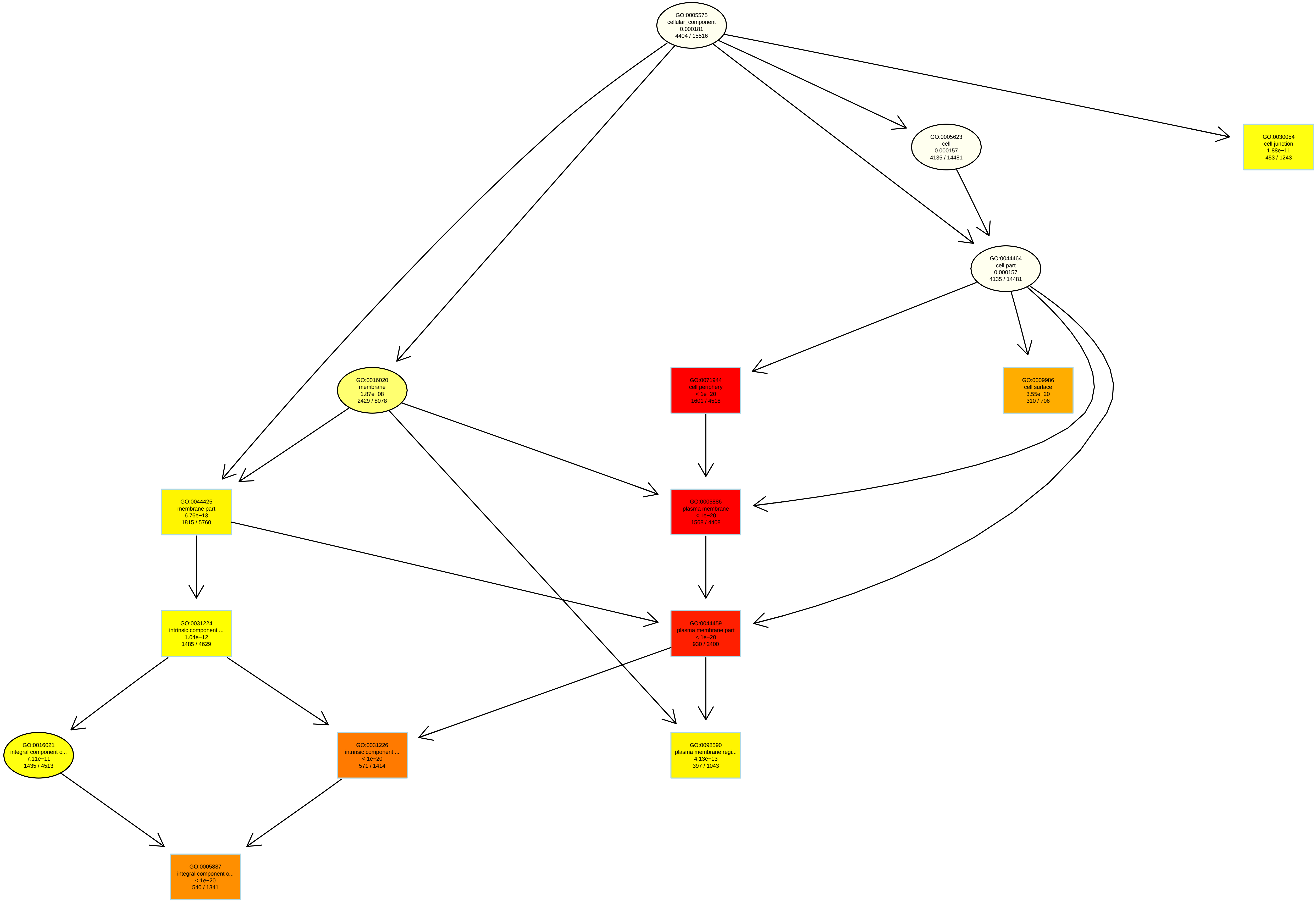
GO:0005575
cellular_component
0.000181
4404 / 15516
GO:0005623
cell
0.000157
4135 / 14481
GO:0030054
cell junction
1.88e−11
453 / 1243
GO:0044464
cell part
0.000157
4135 / 14481
GO:0016020
membrane
1.87e−08
2429 / 8078
GO:0071944
cell periphery
< 1e−20
1601 / 4518
GO:0009986
cell surface
3.55e−20
310 / 706
GO:0044425
membrane part
6.76e−13
1815 / 5760
GO:0005886
plasma membrane
< 1e−20
1568 / 4408
GO:0031224
intrinsic component ...
1.04e−12
1485 / 4629
GO:0044459
plasma membrane part
< 1e−20
930 / 2400
GO:0016021
integral component o...
7.11e−11
1435 / 4513
GO:0031226
intrinsic component ...
< 1e−20
571 / 1414
GO:0098590
plasma membrane regi...
4.13e−13
397 / 1043
GO:0005887
integral component o...
< 1e−20
540 / 1341
